# Evaluating the Feasibility and Participants’ Representativeness of an Online Nationwide Surveillance System for Influenza in France

**DOI:** 10.1371/journal.pone.0073675

**Published:** 2013-09-11

**Authors:** Marion Debin, Clément Turbelin, Thierry Blanchon, Isabelle Bonmarin, Alessandra Falchi, Thomas Hanslik, Daniel Levy-Bruhl, Chiara Poletto, Vittoria Colizza

**Affiliations:** 1 INSERM, U707, Paris, France; 2 UPMC Univ Paris 06, Faculté de Médecine Pierre et Marie Curie, UMR S 707, Paris, France; 3 Department of Infectious Diseases, Institut de Veille Sanitaire (InVS), St Maurice, France; 4 Assistance Publique Hopitaux de Paris, Service de Medecine Interne, Hopital Ambroise Pare, Boulogne Billancourt, France; 5 Institute for Scientific Interchange (ISI), Torino, Italy; University of Zaragoza, Spain

## Abstract

The increasing Internet coverage and the widespread use of digital devices offer the possibility to develop new digital surveillance systems potentially capable to provide important aid to epidemiological and public health monitoring and research. In France, a new nationwide surveillance system for influenza-like illness, GrippeNet.fr, was introduced since the 2011/2012 season based on an online participatory mechanism and open to the general population. We evaluate the recruitment and participation of users to the first pilot season with respect to similar efforts in Europe to assess the feasibility of establishing a participative network of surveillance in France. We further investigate the representativeness of the GrippeNet.fr population along a set of indicators on geographical, demographic, socio-economic and health aspects. Participation was widespread in the country and with rates comparable to other European countries with partnered projects running since a longer time. It was not representative of the general population in terms of age and gender, however all age classes were represented, including the older classes (65+ years old), generally less familiar with the digital world, but considered at high risk for influenza complications. Once adjusted on demographic indicators, the GrippeNet.fr population is found to be more frequently employed, with a higher education level and vaccination rate with respect to the general population. A similar propensity to commute for work to different regions was observed, and no significant difference was found for asthma and diabetes. Results show the feasibility of the system, provide indications to inform adjusted epidemic analyses, and highlight the presence of specific population groups that need to be addressed by targeted communication strategies to achieve a higher representativeness in the following seasons.

## Introduction

Influenza surveillance systems provide essential information to describe the spatiotemporal diffusion of the disease, monitor the incidence through the season and across affected groups, and for the activation of specific response plans and the evaluation of their effectiveness [1], [2]. These systems are traditionally based on national networks of physicians who report cases of patients with influenza-like illness (ILI) and collect samples from a subset of these patients for virological tests, and also on hospital and emergency services [3].

From paper records to electronic systems, developments in modern communication have changed the monitoring systems in the last 30 years, speeding up the transmission of information and facilitating the storage of the data and its access [4], [5], [6]. Additional substantial changes appear nowadays potentially feasible if we consider that digital progress has also changed the way we create and exchange information, shifting the point of view from infrastructures to their users. New technologies have opened the way to real-time, collaborative and mobile mechanisms, allowing a massive amount of digital data to be created, stored, analyzed, modeled, and accessible/shareable on the Web [7], [8]. With much of this data provided directly from the user through digital devices and thanks to the continuously increasing widespread coverage of the Internet access, such digital behaviors also offer the possibility to develop a digital counterpart of the traditional epidemiology [9], [10]. This applies to disease surveillance systems as well, with the development of innovative approaches that are able to detect emerging outbreaks or infer the epidemic activity levels from web search records [11], [12], online news [13], [14], or tweets exchanged by users [15].

Following previous experiences in other European countries [16], our approach leveraged on the social and collaborative aspects of the Web for the setup of an online surveillance system for ILI aimed at gathering data on a large spectrum of information. Not only illness incidence in the community, but also information on health-care usage, contacts with specific classes of individuals (e.g. children, patients, and others), and infection-related attitudes or change of behaviors is collected. To complete the picture on the impact of an influenza epidemic in the general population, we rely on Web-based questionnaires as an appealing alternative option to the traditional methods of data collection based on offline surveys with paper questionnaires and interviews, often requiring high costs in large study populations [9].

The system is called GrippeNet.fr and was launched in January 2012. Participants are volunteers recruited in the general population who register to the project providing general background information and who are then invited to self-report on their ILI symptoms on a weekly basis. While establishing efficient recruitment strategies poses a series of challenges in terms of engagement, it potentially enables to largely increase the coverage in the population. Participatory surveillance has also the great advantage of making it possible to profile the population joining the project, in order to assess the representativeness of the surveillance system and determine risk groups for ILI. Such population may also include individuals who do not seek for health care treatment following infection (an estimated 57% for secondary cases in France [17]). In addition, profiling the population with a large set of indicators is generally not possible in other digital approaches for surveillance, since they rely on the unsupervised collection of information [11], [13], [14], [15], and it may be difficult also in the case of sentinel influenza surveillance because of its protocols and the limited set of information that are routinely collected (age, gender, geography) [18].

Focusing on the pilot season, this work aims to (i) assess the feasibility of the collaborative influenza surveillance system in France, and to (ii) evaluate the representativeness of the GrippeNet.fr population using a set of indicators on geographical, demographic, mobility, socio-economic and health aspects. The goal is to provide useful information for the validation of the study, for the future recruitment of participants in the following seasons, and for identifying possible sample biases to be taken into account into epidemic analyses.

## Methods

### Study Design

GrippeNet.fr is part of a European multicenter study (Influenzanet [16]) for ILI surveillance in the general population through online systems. Such systems were first implemented in Belgium and The Netherlands in 2003 [19], [20], followed by Portugal in 2005 [21], [22], Italy in 2008 [23], the UK in 2009 [24], [25]. France and Sweden joined the system in the 2011–2012 season [26], followed by Spain in the 2012–2013 season [27].

GrippeNet.fr is coordinated by the Sentinel Network team of the National Institute of Health and Medical Research [28] and University Pierre et Marie Curie [29] (also responsible in France for the disease surveillance network of general practitioners (GPs) [30]), and by the National Institute for Public Health Surveillance [31]. Launched on the 25^th^ of January 2012, it consists of a website used to register participants, provide access to participants to their secure and private areas where to fill in the GrippeNet.fr surveys, communicate the results of the study and report on related activities.

During its first season, the project was open to the recruitment of individuals aged ≥18 and resident in mainland France (overseas territories excluded). Recruitment occurred through press releases of the supporting institutions, communications on the Internet, radio, TV, newspapers, and through emails and word of mouth (more information can be found on the webpage ‘GrippeNet.fr in the news’ [32]). A weekly report on the GrippeNet.fr results updated on influenza surveillance and on the participating population was published every Wednesday within the official Weekly Bulletin of the Sentinel Network of GP Surveillance [33], and the Weekly Bulletin of the National Institute for Public Health Surveillance [31].

Participation was voluntary and anonymous. Registration took place on the GrippeNet.fr website where participants could create a user account. A valid email address was required to confirm the registration and avoid unsupervised registration attempts. The email address was also used to maintain a communication channel with each participant as a motivation purpose during the season, as explained in the Data collection subsection. The website contains several pages that are publicly accessible dedicated to the presentation and aims of the project, instructions on how to participate, general information about influenza virus and influenza epidemics and pandemics, and the results of the study provided in real time.

### Privacy and Ethical Approval

This study was conducted in agreement with French regulations on privacy and data collection and treatment and was approved by the Comité consultatif sur le traitement de l’information en matière de recherche (CCTIRS, Advisory committee on information processing for research, authorization 11.565) and by the Commission Nationale de l’Informatique et des Libertés (CNIL, French Data Protection Authority, authorization DR-2012–024).

### Data Collection

Upon registration, the user was asked to complete an intake survey covering demographic factors (age, gender), geographical factors (location of home and work/school expressed at the municipality level), socio-economic factors (household size and composition, occupation, educational level, number of daily contacts with groups of patients, children or elderly, daily transportation means), and health-related factors (height and weight, diet, vaccination status, pregnancy status, smoking habits, major risk conditions, and others). At-risk population was identified as the population eligible for a vaccination voucher, based on the vaccination system for this population group in France (see subsection National Data Sources for more details). The full list of variables for which data was collected in the intake survey is reported in Tables 1 and 2, and in Table S1 in File S1. The survey is available both in English and in the original French version in File S1.

**Table 1:** Socio-demographic indicators gathered in the intake survey and comparison with national statistics.

Indicators	Definition	Mean value (SD) or% in GrippeNet.frpopulation	Mean value or% in Frenchpopulation	p-value	Refs
Gender	Female	65.1%	52.3%	*p*<10^−6^	[35]
Month and year of birth	Age in years	52.1 (14.6)	48.6	*p*<10^−6^	[35]
Main activity	Paid employment	50.9%	48.7%	*p* = 0.040	
	Self employed	4.5%	4.9%		
	Other^1^	44.6%	46.4%		[35]
Level of education	Participant with at least highschool level of educationin [25]–[34] age class(women; men)	95.1%; 96.9%	70.2%; 61.7%	*p*<10^−6^	[35]
	In [35]–[44] age class	93.6%; 94.1%	54.9%; 47.6%	*p*<10^−6^	
	In [45]–[54] age class	83.6%; 87.1%	39.4%; 32.9%	*p*<10^−6^	
	In [55]–[64] age class	81.8%; 71.8%	30.1%; 29.9%	*p*<10^−6^	
Daily contacts with specificgroups of persons	With patients	14.3%	2.2%^2^	*p*<10^−6^	[62]
Main mean of transportation	Car	59.9%	52%	*p*<10^−6^	[63]
	Public transportation	19.7%	28% (metro+train)	*p*<10^−6^	
Pets at home		44.4%	48.7%	*p*<10^−5^	[64]

1 students, home-makers, unemployed, long-term sick-leave or parental leave, retired, others.

2 % of health-care workers in over-18 French population, indicated as a possible match with the % of GrippeNet.fr participants having contacts with patients.

GrippeNet.fr indicators are adjusted by age and gender. In some cases we considered only a partial definition of the variable, with respect to the full definition available in the survey (see File S1) in order to match available national statistics.

**Table 2:** Health-related indicators gathered in the intake survey and comparison with national statistics.

Indicators	Definition	Mean value (SD) or% in GrippeNet.frpopulation	Mean value or% in Frenchpopulation	p-value	Refs
Vaccination against seasonalinfluenza during currentseason (2011/2012)	Vaccinated this year	30.7%†	23%†	*p*<10^−6^	
Vaccination against seasonalinfluenza during previousseason (2010/2011)	Yes	35.0%†	-†		
Taking regular medicationsfor risk conditions	Asthma	5.9%	6.0%	*p = * 0.17	[42]
	Diabetes ([45–74]y; ≥75y)	4.7%; 6.0%	5.8%; 9.6%	*p = * 0.07; *p = * 0.11	[37], [40]
Weight and height	25≤ BMI <30 (women)	21.1%	26.3%	*p = * 4*10^−6^	[39]
	BMI ≥30(women)	9.0%	15.7%	*p*<10^−6^	
	25≤ BMI <30 (men)	30.6%	38.8%	*p*<10^−6^	
	BMI ≥30 (men)	9.5%	14.3%	*p*<10^−6^	
Smoking habits	Women; men	9.5%;11.8%	27.9%; 35.6%	*p*<10^−6^	[41]

† see Table 4 for further breakdown.

GrippeNet.fr indicators are adjusted by age and gender. In some cases we considered only a partial definition of the variable, with respect to the full definition available in the survey (see File S1) in order to match available national statistics.

Participants were also allowed to register and create accounts for other individuals on their behalf, given consent, thus enabling, for instance, to grant access to the project to individuals less comfortable with Internet tools (e.g. in the older age classes). The data was collected only once at the enrollment, however the participant could access her/his intake survey anytime and modify it following any update (e.g. change of residence, of vaccination or pregnancy status). In presence of multiple instances of intake surveys, we considered the last completed.

Each week participants were asked to fill in a weekly symptoms survey, independently of possible ILI symptoms. An automatic email message was sent weekly in the form of a newsletter that contained a reminder to fill in the symptoms survey as well. The newsletter was also meant to keep a high motivation in the participation to the study by providing preliminary results on the analysis of the symptoms reported the week before, a general description of the influenza epidemic situation in France and in Europe, and an entertaining ‘Did you know?’ column about various flu curiosities and historical facts.

The description of the weekly symptoms survey will be reported elsewhere, as here we focus on the representativeness of the project participants and will analyze exclusively the data collected through the intake survey. A final feedback survey was conducted during spring 2012, after the closure of the data collection campaign, to gather information on the perception of users regarding their participation to the study.

### Inclusion Criteria

All questionnaires filled in between the 25^th^ of January 2012, launch date of the project, and the 29^th^ of April 2012, closure date of the first online surveillance season, were considered in the analyses, covering a total of 14 weeks. To prevent that a short-term participant may bias our results [34], we include in our sample only those participants who completed an intake questionnaire and at least three symptoms surveys during the season [22] – such individuals being referred to, hereafter, as *active participants* (in the following we will equally refer to GrippeNet.fr population or Grippenet.fr active population to indicate the population of active participants). No additional specific constraint was required on the frequency of compiling the symptoms survey during the 14 weeks of surveillance of the project. We tested these inclusion criteria and performed a sensitivity analysis of our results by imposing that participants would fill at least 3 symptoms surveys, and at least one symptoms survey every calendar month since they joined the study. This prevents us to consider participants who may have filled in the symptoms surveys in three consecutive weeks and then dropped out. The representativeness of participants who did not specify their age and/or gender in their intake questionnaire cannot be assessed, so we excluded those participants from the analyses.

### National Data Sources

The study required gathering data from a number of socio-demographic datasets and health datasets that are described hereafter.

The demographic and socioeconomic data of the French population were provided by the French National Institute of Statistics and Economic Studies (INSEE). In absence of data for the year of 2012, we relied on the most recent available sources: age-gender composition updated to January 1, 2010; household size and composition as of 2009; employment data updated to December 31, 2010; education data as of 2010; city size as of 2009. All data concern only mainland France [35].

In the following we indicate as French adult population or general population, the population of mainland France being 18 years old or older.

In France, the at-risk population who is recommended for vaccination by public health authorities includes the following categories: individuals aged over 65, pregnant women, obese individuals, and individuals with chronic health conditions (diabetes, pulmonary, kidney or heart disease, and others). Vaccination for these classes of individuals is provided by the national health insurance. More in detail, the system relies on mailing vaccination vouchers to individuals who comply with the above definitions (except pregnant women and obese individuals who are not identified by national health insurance). Vouchers can be used in pharmacies and medical practices to receive the influenza vaccination for free. The used vouchers are then mailed back from pharmacies and medical practices to the national health insurance main office for reimbursement purposes. The percentage of individuals who have been recommended for vaccination in French population was estimated through the number of vouchers sent by the national health insurance in the fall 2011, within the French population registered to the national health insurance (data of the CNAM [36]). Taking into account that this population represented 85% of the overall French population [37] and assuming that the percentage of at-risk population is similar in the population of the CNAM and the overall French population we can calculate the percentage of the French population below 65 years of age and registered to the national health insurance who received a voucher, thus representing the estimated at-risk population in France aged <65 years.

The data on influenza vaccination coverage in the French general population was provided by two different sources: the Caisse nationale d’assurance maladie des travailleurs salariés (CNAMTS, French National Health Insurance Fund for Employees) for 2010–2011 for several age groups [38] and 2011–2012 for age groups over 65 years old in the general population, and for at risk population in the <65 years classes [36]; the Groupe d’Expertise et d’Information sur la Grippe (GEIG, French Group of Expertise and Information about Influenza) for 2011–2012 for age groups under 65 years old (data obtained through a phone-based post-epidemic survey conducted yearly by a private independent organization from a representative sample of the French population over 15 years).

Data on body mass index (BMI) were provided by the ObEpi study for 2012 [39]. Data on diabetes was obtained from the CNAMTS through the Ecosante database for 2011 in mainland France [40]. Incidence rates were calculated with 2010 demographic data obtained from INSEE. Data on smoking was provided by the Institut national de prévention et d’éducation pour la santé (Inpes, French national institute for prevention and health education) [41]. Data of asthma prevalence were issued from a previous study [42], using the figure corresponding to the definition “individuals who had an asthma crisis in the last 12 months and/or who currently have a treatment for asthma”, as we consider that the question of the intake questionnaire identified individuals who currently take a treatment, and individuals who take a treatment when they have an asthma crisis.

Commuting data across the 22 regions of mainland France was obtained from the data set provided by INSEE for 2009 [43], the most recent source for commuting. The original data provides the number of daily commuters from a given municipality of origin (corresponding to the location of residence) to a given municipality of destination (corresponding to the location of school or workplace). All individuals aged more than 3 years old are recorded in the data set. The original data is then mapped to the region level in order to obtain the number of commuters who live in a given region and daily commute for educational or professional reasons to another region, for any pair of regions in France.

### Data Analysis

The representativeness of the GrippeNet.fr population was assessed through the comparison of its characteristics with the ones of the general French population, considered as the reference population, with χ^2^-test for non-continuous sociodemographic variables, and Student’s *t*-test for mean comparisons. All comparisons used bilateral tests and a 5% cutoff point. Statistical analyses were performed using the R software version 2.13.2 (R Development Core Team, R Foundation for Statistical Computing, Vienna, Austria, http://www.r-project.org).

Apart geographical and demographic characteristics, a standardized analysis on age group and gender [24] was done on the other variables, considering the following age categories : 18–29, 30–39, 40–49, 50–59, 60–69, ≥70.

The commuting data, extracted from the census and from the GrippeNet.fr population, were analyzed in terms of networks of nodes and links [44], [45], where nodes represent the regions and a directed link from a region of origin O to a region of destination D is drawn if there exist at least one individual resident in O and daily commuting to D. A weight 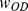 was also assigned to each link to indicate the number of commuters on that connection. The census datum was adjusted for possible discrepancies in the geographic distributions of GrippeNet.fr population with respect to the national data. Then, the most relevant backbone of the commuting network was extracted through the disparity filter algorithm [46] to be compared with the GrippeNet.fr network. The details of the analysis are reported in File S1.

We also calculated the probability to observe the directed links in the GrippeNet.fr commuting, given the probability that an individual living in region O would commute to region D and the sample of the GrippeNet.fr participants in region O (see File S1). These probabilities can then be directly compared to the occurrence of the commuting links in the GrippeNet.fr sample.

## Results

### Descriptive Analysis

The number of participants reached about 3,500 in 5 weeks and continued to increase throughout the season (see Figure S1 in File S1). Between the 25^th^ January and the 29^th^ April 2012, 3,936 persons filled at least one intake survey, and a total of 29,843 symptoms surveys were completed, for an average of 2,130 symptoms surveys per week.

Based on the inclusion criteria, we selected 3,055 active participants. 11 persons did not mention their age and/or gender and were excluded from the analyses. 3,044 individuals were therefore considered in the following analyses, corresponding to 77.3% of the entire sample. Among them, 2827 (92.9%) had a single membership account, 190 (6.2%) belonged to a household (i.e. multiple) account with 2 active participants, 27 (0.9%) with 3 active participants. Active participants filled in between 3 and 28 symptoms surveys, with a median number per active participant equal to 10 (Q1 = 7, Q3 = 11).

Users’ satisfaction concerning their participation to the project was in general very high. Only 0.9% of the active participants who completed the feedback survey during summer 2012 (for a total of more than 1,900 respondents) affirmed that they were not willing to participate to the following season. Moreover, only 1.4% of them declared that the weekly questionnaire takes too long, whereas 87.7% of them found the website easy to access and did not find any difficulty in performing the task. General interest to the project beyond the data contribution was also observed, with the majority of responders declaring that they frequently read the newsletter sent every week, and 40.9% of them visiting the website for further information, besides the access to the surveys.

### Geographical and Demographic Characteristics

**Figure 1:**
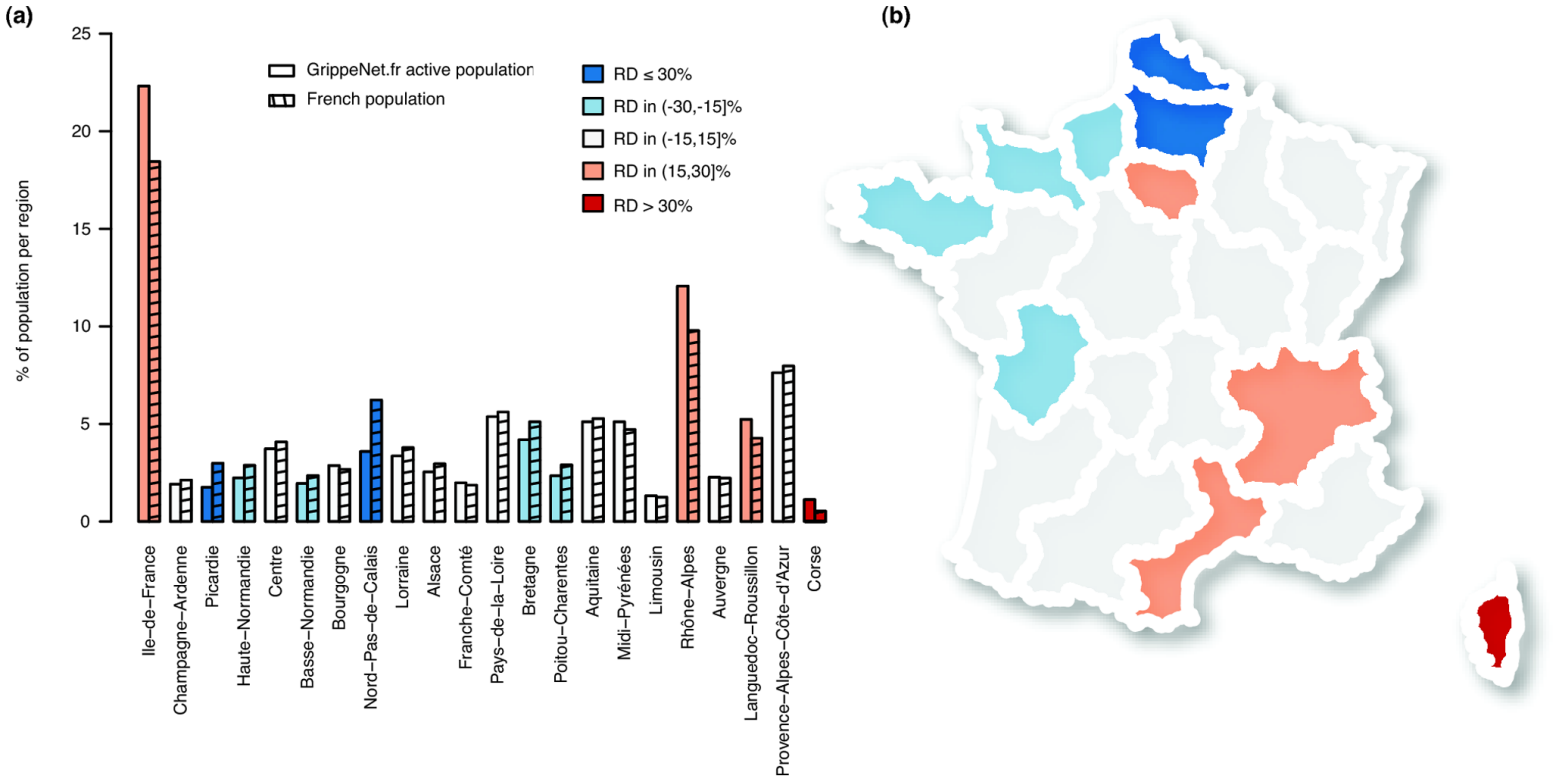
Distribution of the GrippeNet.fr active participants per region. a) Comparison with the distribution of the over-18 French population per region. b) Relative difference (RD) between regional distribution of GrippeNet.fr population and French population.

Our study reached a coverage of 6.2 individuals per 100,000 inhabitants and participation was widespread in the country, with active participants coming from all 96 mainland French departments, for a total of 1,783 distinct municipalities participating to the project (see Figure S2 in File S1). The geographic distribution of the participant population in French regions is statistically different from the distribution of French population (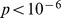), with, e.g., the largest overrepresentation in the Corse region, and a high underrepresentation in the regions of Nord-Pas-de-Calais and Picardie. We note that, with the exception of the Ile-de-France, under-representation is generally localized in northern regions (see Figure 1). The majority of the regions (12 out of 22, 54%) has however a relative difference between regional distribution of GrippeNet.fr population and French population contained in the range [−15%;+15%].

Inhabitants of big cities are more likely to participate to GrippeNet.fr than those of smaller cities; 55.3% (95% C.I. = 53.6%–57.1%) of active participants coming from cities with population >10,000 inhabitants, against 49.6% obtained for the French population (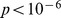).

**Figure 2:**
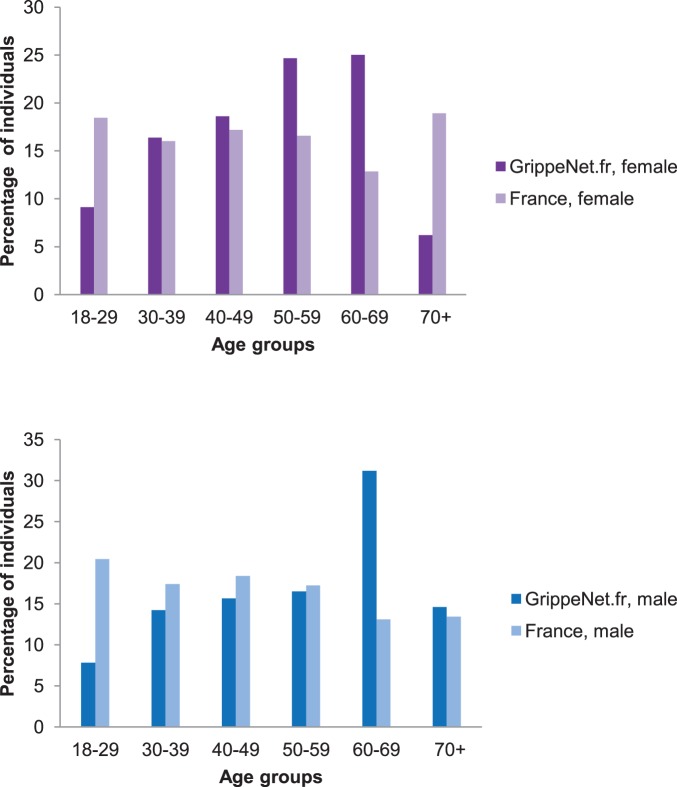
Distribution by age and gender of the GrippeNet.fr active participants. Comparison of the distribution by age class of the female (panel a) and male (panel b) population between the GrippeNet.fr sample and the over-18 French population.

The GrippeNet.fr population was on average older than the French population, with a mean age for active participants being equal to 52.1 years old (median equal to 53.5; 95% C.I. = 23.4–80.7) against 48.6 years old in France (once individuals under 18 are not taken into account) (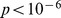). A larger proportion of females (65.1%, 95% CI = 63.5%–66.8%) participated to the study, with respect to 52.3%, the baseline value (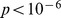). All age classes were represented in the sample, however a significant difference between the repartition in age and gender of active participants and French population is observed (see Table 3) (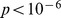). Individuals aged <30 were found to be underrepresented in both female and male populations (as well as the 70+ class in the female population), whereas the opposite occurs for the age class [60–69]. A large over-representation was also observed in the female [50]–[59] class, contrary to the results found for male individuals in the same age group (Figure 2).

**Table 3:** Overall comparison of *GrippeNet.fr* population with French population, for different age and gender groups (all differences are significant,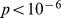).

	Age	*GrippeNet.fr* population (%)	French population (%)
**Female**	18–29	5.9	9.6
	30–39	10.7	8.4
	40–49	12.1	9.0
	50–59	16.1	8. 7
	60–69	16.3	6.7
	≥70	4.0	9.9
	**Total**	**65.1**	**52.3**
**Male**	18–29	2.7	9.7
	30–39	5.0	8.3
	40–49	5.4	8.8
	50–59	5.8	8.2
	60–69	10.9	6.3
	≥70	5.1	6.4
	**Total**	**34.9**	**47.7**

### Mobility Features

Among the active participants, 1,078 individuals (61.6% of the studying/working GrippeNet.fr population) provided information on their school/work locations, specifying the corresponding municipality codes. Once the data was analyzed at the regional level, most of the participants declared to commute within the administrative region of their residence. The ratio of the cross-region commuters over the within-region commuters is 5.4% [0%,19%], very close to the corresponding value for census data (3.7% [1%,10%]).

GrippeNet.fr commuting network is composed of 22 nodes as in the census commuting network, since all regions are covered by the study. Only 28 links over the 462 of the census network are however represented in the participant population. Four regions (Corse, Limousin, Poitou-Charentes, Bourgogne) are disconnected from the rest of the network, as they do not have either incoming or outgoing commuters. If we compute the relative difference of the distribution of incoming commuters (or outgoing commuters) of the sample vs. census of a given region, we find quite a large disagreement between the two datasets, with only one region (Provence-Alpes-Côte-d’Azur) and two regions (Haute-Normandie and Centre) lying in the interval [−15%,15%] for in-commuters and out-commuters, respectively (see Figure S4 in File S1 for more details).

**Figure 3:**
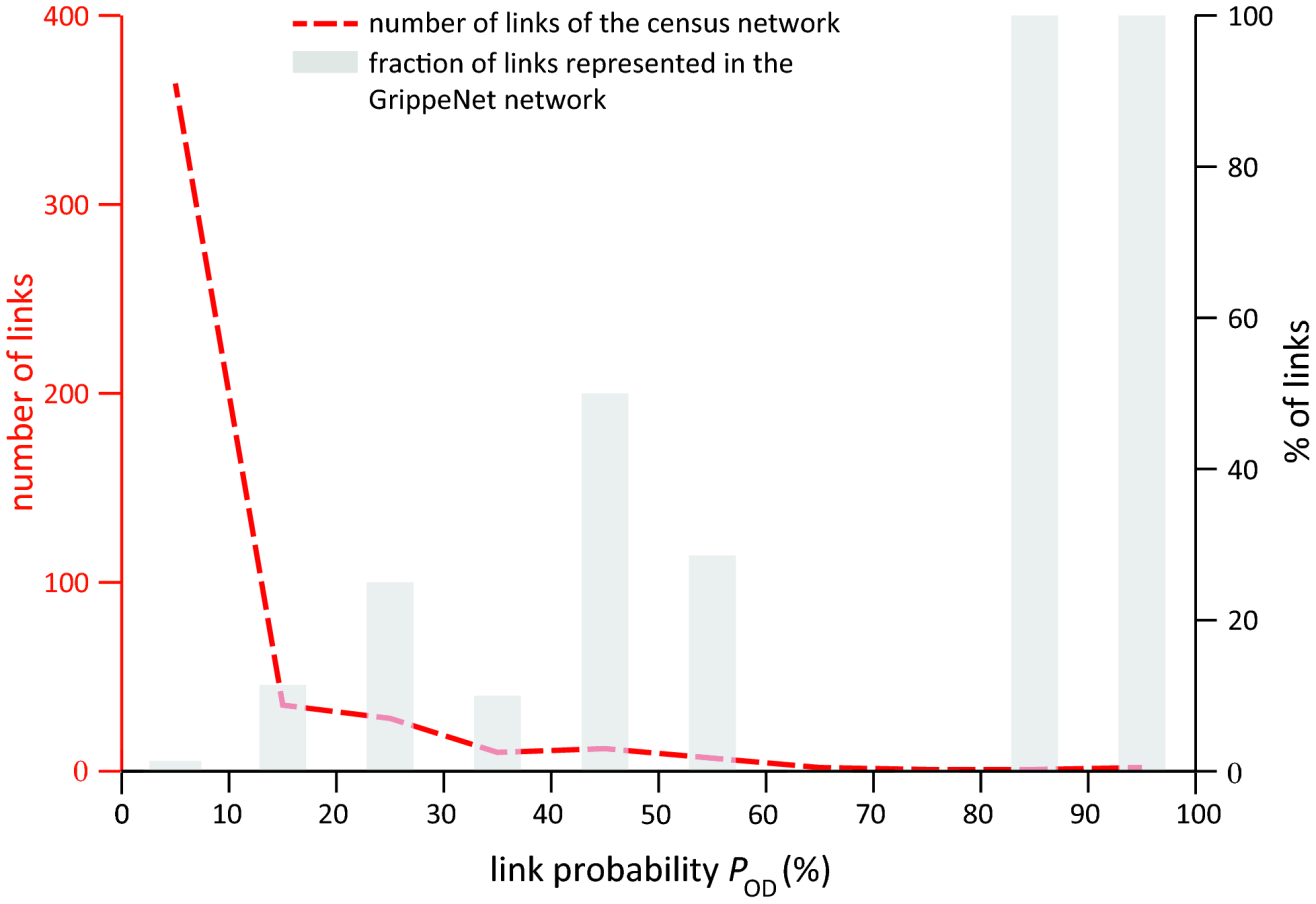
Percentage of commuting links recovered by the GrippeNet.fr population. The percentages are presented as a function of the probability of occurrence of the link. The bar plot shows the percentage of links of the census network represented in the GrippeNet.fr sample per probability interval. The probability of the link is a measure of the likelihood that a GrippeNet.fr participant would commute, taking into account the probability of commuting for an individual in the French population and the GrippeNet.fr sample. The red curve indicates the number of links in the French census network with a given probability of occurrence.

Going beyond the comparison between absence or presence of the commuting links, we tested whether the GrippeNet.fr sample well reproduces the largest flows of mobility within the country, given the large variability of commuting fluxes of the French census commuting network (see Figure S3 in File S1) [47]. Half of the census commuting links having a probability larger than 60% are represented in the GrippeNet.fr population (Figure 3), despite that they are very few (less than 1% of the total). A smaller representation (less than 30% of the links) is obtained for the links having probabilities of occurrence in the range [20], [60]%, whereas links with probabilities smaller than 10% are almost not found in the GrippeNet.fr commuting network.

**Figure 4:**
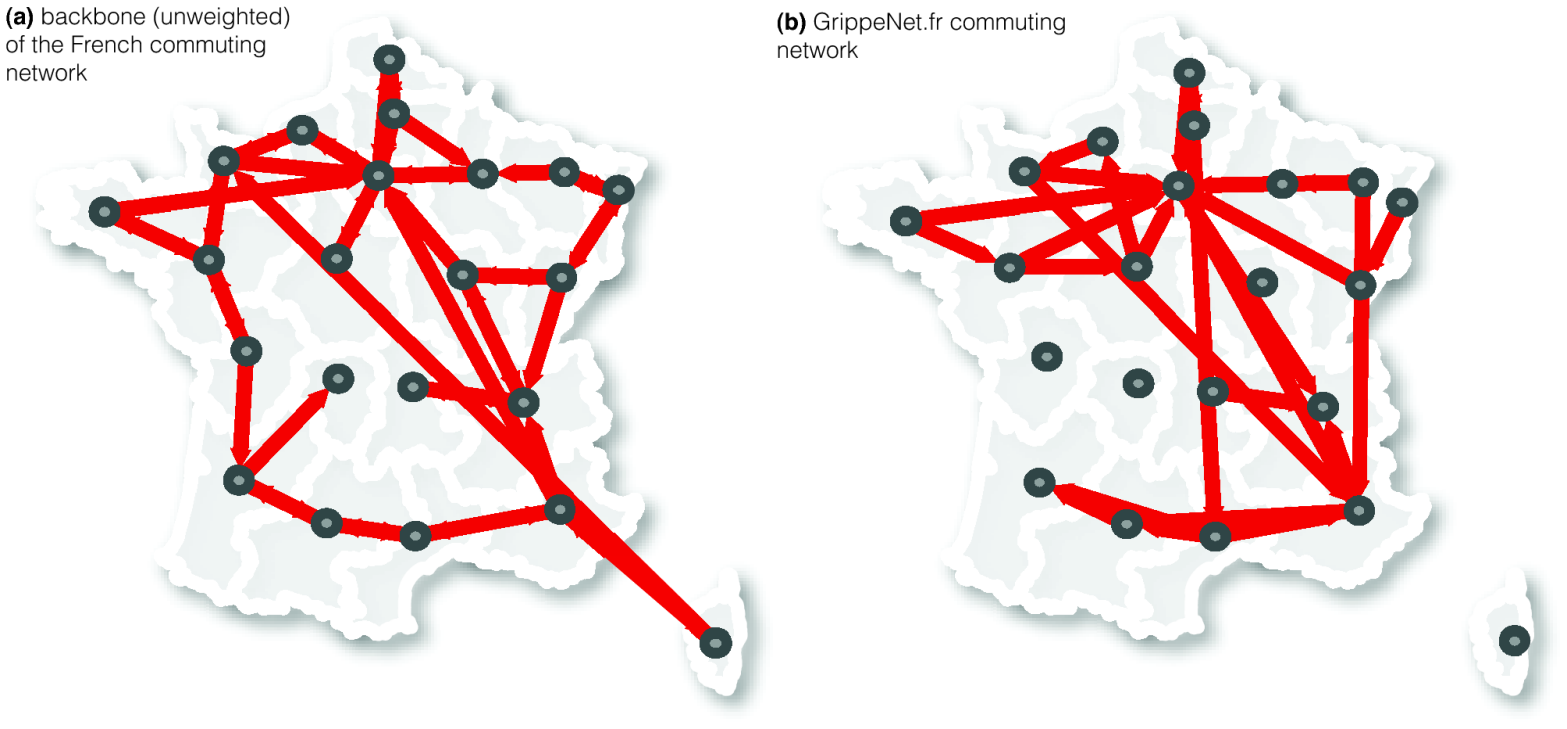
Comparison of the commuting network structure between the GrippeNet.fr and the French population. a) Backbone of the census network as extracted by the disparity filter algorithm. b) GrippeNet network. Arrows represent directed links of commuters from a region of origin to a region of destination. The backbone of the census network has 49 links and includes 17 of the 28 links of the GrippeNet network.

In addition to the most probable links, we can also test the representativeness of the statistically most relevant links forming the backbone of the commuting network extracted with the disparity filter algorithm. GrippeNet.fr commuting network is able to capture some of the relevant features of the French commuting pattern at the level of regions (Figure 4). The centrality of Île-de-France is recovered, as well as the strong commuting pattern among the southern regions, and the connections between Northern regions and Southern regions. A larger discrepancy is instead observed in the absence of connections between the Corse region and the rest of the country.

### Socio-economic Factors

Active participants more often had at least a high school level of education (equivalent to “baccalauréat”), ranging from 71.8% (95% C.I. = 65.8%–77.9%) in the [55]–[64] age group (vs. 29.9% in the general population) up to 96.9% (95% C.I. = 94.9%–99.0%) in the [25]–[34] (vs. 61.7%) among males (see Table 1). Analogous results were found in the female sample (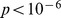).

A significant difference (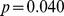) was found in the occupation profile of the GrippeNet.fr population with respect to the general population, with 50.9% (95% C.I. = 49.1%–52.7%) vs. 48.7% of paid employed individuals, and 4.5% (95% C.I. = 4.3%–4.6%) vs. 4.9% of self-employed individuals, respectively.

### Health Factors and Vaccination

The prevalence of overweighted and obese individuals is significantly lower in GrippeNet.fr population than in French population, for men (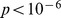) and for women (respectively 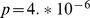
and 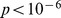) (Table 1).

No significant difference was found between GrippeNet.fr and French populations for diabetes prevalence in the age classes of [45–74] 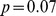 and of ≥75 (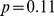), however no national data is available for comparison in individuals between 18 and 45 years old (see Table 2).

Among GrippeNet.fr participants, 5.9% (95% C.I. = 5.1%–6.7%) declared that they take regular medication for asthma, while the prevalence for asthma is 6% in French adults (non significant difference,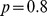).

Vaccination coverage to seasonal influenza in the 2011–2012 season was higher in our sample (Table 4) (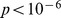). The difference between the two populations is greater in younger age groups (12.6%, 95% C.I. = 10.0%–15.2% vs. 6% in [25]–[34] age group) and decreases when the age group increases (63.1%, 95% C.I. = 59.6%–66.6% vs. 55.2% in individuals older than 65). A comparable difference is observed for individuals older than 65 in the 2010–2011 season (62.8%, 95% C.I. = 59.3%–66.4% vs. 53.8%).

**Table 4:** Percentage of seasonal influenza vaccination coverage, comparison between *GrippeNet.fr* and French population (all differences are significant, 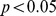).

	Vaccination in2010–2011 season		Vaccination in2011–2012 season	
Age group	GrippeNet.fr	French population(CNAMTS)	GrippeNet.fr	French population(GEIG)
** 25–34**	20.6		12.6	6
** 35–49**	27.4		21.9	10
** 50–64**	35.4		30.4	21
** >65**	62.8	53.8	63.1	55.2^1^
** Total**	**35.0**		**30.7**	**23**

1 For this age group, the figure was obtained from Ref. [36].

In the fall 2011, within the French population registered to the national health insurance, 2,074,437 vouchers were sent to at risk individuals aged <65, representing 4.7% of the French population below 65 years of age and registered to the national health insurance. 819,841 of them got vaccinated (39.5%). 10.7% (95% C.I. = 9.5% –12.0%) of GrippeNet.fr participants under 65 received a vaccination voucher in the fall 2011, thus leading to an overrepresentation of the at-risk population aged <65 years in our sample (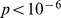). This particular group of the population was also more likely to be vaccinated in the GrippeNet.fr population with respect to the French population (vaccination rate, 53.0%, 95% C.I. = 46.8% –59.3% vs. 39.5%, 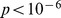).

## Discussion

The GrippeNet.fr surveillance system was launched during the 2011/2012 season to serve a surveillance function complementing the traditional disease surveillance [30], [48] with a rapid, flexible, and collaborative Web approach. This first study aimed to assess the feasibility of the approach regarding the participation of the French population, following other European countries, and to evaluate the representativeness of the population participating to the project.

Almost 4,000 individuals registered to the project during its first season, from all regions of the country. These results on the voluntary uptake of a newly developed project are encouraging, especially if we note that no massive communication campaign was put in place during the pilot season. Dropouts or highly discontinuous participation was very low and limited to less than 23% of the total number of registered individuals. The remaining 77% represents the set of active participants (considered here for the analysis of the representativeness), displaying an active participation rate within the sample consistent with previous experiences in other countries (from 69% in Portugal to 87% in The Netherlands in the 2006/2007 influenza season [22]). With one season only of data collection campaign, the system reached a national participation of 6.2 active participants per 100,000 inhabitants, attaining the same levels of participation of countries running the online surveillance system since a longer time (see Table S2 in File S1). The number of participants is clearly a critical aspect for obtaining accurate estimates that can be used to run epidemiological surveillance and cohort studies. With the next challenge being to increase the participation rate of the population, it is reassuring to note that >99% of the participants who responded to the satisfaction survey conducted at the end of the first season confirmed their willingness to continue their participation in the following seasons.

Ensuring a satisfactory level of representativeness of the sample compared to the general population is however crucial to allow further analyses. The GrippeNet.fr population is not representative in terms of age and gender, with a large overrepresentation of women, and an underrepresentation of the youngest and oldest age classes. This is most likely due to two factors: the non-representative nature of the Internet population, thus translating into coverage biases, and the self-selection of participants, also called the ‘volunteer effect’ [49]. While many studies are advocating the use of the Internet and of other digital tools to collect valuable information about disease and health dynamics (see e.g. [10] and references therein), the use of the Web and the adoption of social media services may strongly vary among population groups [50]. In France more than 64% of the households had an Internet access in 2010 [51], showing a dramatic dependence on age (93.3% in [15]–[29] age class; 85.7% in [30]–[44]; 44.3% in [60–74], and only 8.3% for individuals aged 75+), and on education (29.1% for individuals with no qualifications vs. 91.1% in the highly qualified population). This may therefore explain the underrepresentation of both the elderly class and the class of individuals with low education level in the GrippeNet.fr population, a characteristic commonly found in web-based surveys [19], [22], [52], [53], [54]. To achieve a better representativeness of individuals in these classes, the system will need to design targeted communication campaigns for these groups and facilitate the accessibility to the project. The latter could be achieved along two directions. On one side, it could aim at increasing availability and improving physical accessibility promoting digital education (see Ref. [55] as an example set in a European country with low Internet penetration); on the other, it could focus on the improvement of the graphical user interface of the online system to make it easier and more accessible for individuals not familiar with the Web and its applications.

Two discrepancies however arise that are not consistent with the Internet access statistics – namely, the strong underrepresentation of young individuals (aged [18]–[29]) and a strong overrepresentation of the population in the [60–69] age class. These features are common to both males and females, with the female sample also showing an overrepresentation in the [50]–[59] age class, similarly to the results found in other European countries [24], [34], [56]. Internet and social media usage are widespread among the younger portion of the population, however our results seem to indicate a low interest towards influenza-related topics that may affect their voluntary participation to the project. Different findings may be obtained in the case of similar web-based scientific surveys focusing on other topics than influenza (e.g. food behavior, diet and health [57]), and will be the object of future work. These discrepancies should be considered when planning the future developments of the system, e.g. by considering the creation of increasingly engaging mobile applications for data collection.

An opposite behavior in the self-selection bias may be at play in the [60–69] age class, as these individuals may be more interested in influenza-related topics, because for instance they may be more frequently concerned by influenza and its complications. We assessed that our sample is indeed non-representative with respect to risk status, with an over-sampling of the population in this group.

The substantial imbalance in the participation per gender is similar to the one observed in the UK [52], and more pronounced than the discrepancy observed in the Netherlands [19]. Evidence suggests that women are on average more interested in health-related topics and also exhibit a more active information-seeking behavior [58], [59]. For these reasons they appear to be more likely to volunteer participation in health questionnaires.

Once the non-representative nature of the GrippeNet.fr population in terms of age and gender is discounted, the mobility of participants well represents the ratio between within-region and across-region commuters observed in the country, a feature that has important implications for the spatial spread of influenza across the country. The sampled commuting network reproduces half of the links with higher probability of occurrence and includes the majority of the statistically relevant connections of the census commuting network. Discrepancies are most likely due to the limited sample size, given that commuting across distant regions is not highly probable, and we expect an improvement in the comparison of the mobility behavior with higher participation rates.

The GrippeNet.fr population is characterized by a higher vaccination rate with respect to the French population, and it includes a larger number of at risk individuals in the <65 class. We expect the same mechanism already discussed for the larger representativeness of the [60–69] age class to be at play. Surprisingly, the prevalence of other conditions, such as diabetes and asthma, is however not statistically different from the values observed in the French population. Given that at risk individuals under 65 years of age are defined by the presence of chronic pathologies, we expect that the overrepresentation of this category is due to other conditions different from asthma and diabetes. However no data at the national level is available to test this hypothesis. GrippeNet.fr participants are less frequently found to be overweight and obese than the French population, reflecting a higher concern in health-related issues, as well as a higher socio-educational level of participants [39].

The overrepresentation of the vaccinated class is a critical point for the evaluation of the surveillance system in terms of incidence and risk factors. Adjusting for age, gender, and vaccination status would require an age/gender classification of vaccinated individuals in the general population that is not yet available in France, whereas it is routinely collected in other European countries [60], [61]. While in principle the inclusion of other parameters should be considered as well in the reweighting procedure (such as e.g. chronic diseases, educational level), detailed cross-reference data at the national level are lacking, and moreover this strategy would face sample size issues in underrepresented strata of population. It is important to note however that for other countries ILI incidence trends based on similar systems as GrippeNet.fr associate well with sentinel GP data, and in some cases also with Google Flu Trends data, without correcting for the unrepresentativeness of their population [19], [22], [34], [56].

The online surveillance system is open to all individuals resident in France. Besides the demographic and socio-economic indicators used, we may expect that individuals belonging to minorities or ethnic groups, or foreigners living in France were underrepresented in this study. For ethical reasons, no questions were asked to evaluate the representativeness of the population on those criteria.

As a sensitivity analysis, we have modified the inclusion criteria to test the robustness of our results with respect to the definition of active participants adopted, and imposed a minimum frequency between consecutive symptoms surveys filled (at least one questionnaire per month, between the month of registration and the ending month of the study, April). 2,560 individuals (84% of the total) comply with the modified inclusion criteria. Re-analysis of all the data based on the more restrictive inclusion criteria disclosed no material differences in the interpretation of the results presented in the study and no change in the statistical significance of the results.

### Limitations

One of the potential limitations of the study is the possible lack of accuracy in the data reported by third parties. GrippeNet.fr indeed allows participants to include multiple individuals under the same account, where one person who is the account manager fills in the data for all other account members. This is very convenient in a family setting, for example, where one member of the family fills in the information for the other members, or to include respondents who have limited computer expertise or have no access to the Internet. It is not possible to directly explore the accuracy of the information reported for third parties, however we could in principle test the robustness of the results obtained from the whole sample with respect to the subsample of accounts with multiple memberships.

Our sample of active participants was composed by a vast majority of individuals having a single membership account, with only 7.1% of the active participants belonging to multiple members accounts. The limited size of the sample with multiple members does not allow us to perform statistically significant tests after the pilot season, and we plan to investigate further this aspect in the following years after having reached a larger participation rate. We note however that these biases may be less important when completing the intake survey and may play a more important role in the case of the symptoms survey, where a full list of questions on symptoms, details of occurrence, and actions taken are posed to the participants. We will further investigate this aspect in future work.

## Conclusions

The results of the pilot season confirm the feasibility of the introduction in France of participatory ILI surveillance based on online systems, with participation rates comparable to previous experiences in other countries. Once adjusted on age and gender, the GrippeNet.fr sample is statistically representative of the French population for diabetes and asthma prevalence. The classes of individuals at risk, vaccinated individuals, or with a higher level of education are however all oversampled. These results need to be taken into account to correctly interpret epidemic data and also to plan changes and updates in future seasons. Targeted strategies for communication and the organization of specific activities can be informed by the findings of this study to increase coverage and participation rates, as well as to improve physical accessibility and easiness of use of the platform. Finally, it would be important to assess the generalizability of our results to the other European countries participating in the Influenzanet project, for the validation of the system across countries. This multi-country assessment may also provide useful indicators for further improvements in participation and coverage at the national level, learning from other countries’ experiences.

## Supporting Information

File S1 The file contains the intake survey (French and English versions), additional descriptive indicators non-comparable with national data, a description of the methodology used to analyze the commuting networks and corresponding supporting additional results, and the coverage of partner projects of GrippeNet.fr in other European countries.
(PDF)
