# Supplementary material for: Evaluating the Feasibility and Participants’ Representativeness of an Online Nationwide Surveillance System for Influenza in France
Source: PLoS One. 2013 Sep 11;8(9):e73675. doi: 10.1371/journal.pone.0073675 (PMC3770705; doi:10.1371/journal.pone.0073675)
Supplement: File S1 — The file contains the intake survey (French and English versions), additional descriptive indicators non-comparable with national data, a description of the methodology used to analyze the commuting networks and corresponding supporting additional results, and the coverage of partner projects of GrippeNet.fr in other European countries. (PDF) [file pone.0073675.s001.pdf]

Evaluating the feasibility and participants' representativeness of an online nationwide surveillance system for influenza in France

Marion Debin[1,2], Clement Turbelin[1,2], Thierry Blanchon[1,2], Isabelle Bonmarin[3], Alessandra Falchi[1,2], Thomas Hanslik[1,2,4], Daniel Levy-Bruhl[3], Chiara Poletto[1,2,5], Vittoria Colizza[1,2,5]

1) INSERM, U707, Paris, France
2) UPMC Université Paris 06, Faculté de Médecine Pierre et Marie Curie, UMR S 707, Paris, France
3) Department of infectious diseases. Institut de Veille Sanitaire (InVS), St Maurice Cedex, 94415, France
4) Assistance Publique Hopitaux de Paris, Service de Medecine Interne, Hopital Ambroise Pare, Boulogne Billancourt, France
5) Institute for Scientific Interchange (ISI), Torino, Italy

Supporting Information File S1

1. GrippeNet.fr intake survey (English translation of the original survey).

Mandatory questions are followed by a *.
Where the options is “pick only one” (e.g. Intake Q1) we used filled bullet points; where it is “pick all that apply” (e.g. Intake Q5) we used open bullet points.

Intake Q0
For whom are you filling this survey in?*

- Myself
- A member of my household
- Someone else

Intake Q0b
I hereby certify that I have explained to this people the content and implications of this study, and obtained his free consent to participate. This people gave me the authorisation to answer to his questionnaires.*

- Yes
- No

If « No », it is not possible to fill in the questionnaire, or to answer to other questionnaires. If « Yes », the following text appears : « Please answer to all these questions as if you were this person ».

Intake Q1
What is your gender?*

- Male
- Female

Intake Q2
What is your date of birth (month and year)?*
If the date of birth indicates that the participant is under 18 , it is not possible to fill in the questionnaire, or to answer other questionnaires.

Intake Q3
What is your home postal code?*
This information is not registered in the database, and only allows answering easily to the next question.

Intake Q3b
What is your home municipality?*
Drop-down list thanks to the answer of the question Q3.

Intake Q4
What is your main activity?*

- Paid employment, full-time
- Paid employment, part-time
- Self-employed (businessman, farmer, tradesman, etc)
- Attending school/college/university
- Home-maker (e.g. housewife)
- Unemployed
- Long-term sick-leave or parental leave
- Retired
- Other

Intake Q4b (if Yes to Q4)
What is your school/college/workplace postal code (where you spend the majority of your working/studying time)?

- XXXX
- I don't know/can't remember
- Not applicable (e.g. don't have a fixed workplace)

This information is not registered in the database, and only allows answering easily to the next question.

Intake Q4c
What is your school/college/workplace municipality?
Drop-down list thanks to the answer of the question Q3.

Intake Q4d (if "Yes, Paid employment full time", or "Yes, paid employment part time" to Q4)
Which of the following descriptions most closely matches with your main occupation?

- Professional (e.g. manager, doctor, teacher, nurse, engineer)
- Office work (e.g. admin, finance assistant, receptionist, etc)
- Retail, sales, catering and hospitality and leisure (e.g. shop assistant, waiter, bar-staff, gym instructor etc)
- Skilled manual worker (e.g. mechanic, electrician, technician)
- Other manual work (e.g. cleaning, security, driver)
- Other

Intake Q4e (to be asked for those aged 16 and over)
What is the highest level of formal education qualification that you have? If you are still in education, then please tick this box, with the appropriate highest level that you have already achieved.

- o I have no formal qualifications
- o GCSE's, O'levels, CSEs or equivalent
- o A-Levels or equivalent (e.g. Highers, NVQ Level3, BTEC)
- o Batchelors Degree (BA, BSc) or equivalent (e.g. HND, NVQ Level 4)
- o Higher Degree or equivalent (e.g. Masters Degree, PGCE, PhD, Medical Doctorate, Advanced Professional Awards)
- o I am still in education

Intake Q5
Do you have contact with any of the following during the course of a typical day?* (Select all options that apply, if any)

- o More than 10 children or teenagers over the course of the day
- o More than 10 people aged over 65 over the course of day
- o Patients
- o Groups of people (more than 10 individuals at any one time)
- o None of the above

Intake Q6
INCLUDING YOU, how many people in each of the following age groups live in your household?*
Drop down menus for each of:
I live alone
0-4 years
5-18 years
19-44 years
45-64 years
65+ years

Intake Q6b (If any in household are aged 0-4, including participant)
How many of the children aged 0-4 in your household go to school or day-care?

Intake Q6c (if answer to question Q6b is not 0)
Where do those children go during the day?

- School
- Nursery Assistant
- Joint custody
- Nursery
- Kindergarten
- Family day-care home
- Day-care

Intake Q7
What is your main means of transportation?*

- Walking
- Bike
- Motorbike/scooter
- Car
- Public transportation (bus, train, tube, etc.)
- Other

Intake Q7b
On a normal day, how much time do you spend on public transport (bus, train, tube, etc)?*

- No time at all
- 0-30 minutes
- 30 minutes – 1.5 hours
- 1.5 hours – 4 hours
- Over 4 hours

Intake Q8
How often do you have common colds or flu-like diseases?

- Never
- Once or twice a year
- Between 3 and 5 times a year
- Between 6 and 10 times a year
- More than 10 times a year
- I don't know

Intake Q9a
Did you receive a vaccination voucher for influenza, this year? *

- Yes
- No
- I don't know/can't remember

Intake Q9b
What is your height (in centimeters)?

Intake Q9c
What is your weight (in kilograms)?

Intake Q10
Did you receive a flu vaccine this autumn/winter season (since last September)?

- Yes [go to questions Q10b and Q10c]
- No [go to question Q10d]
- I don't know/can't remember

Intake Q10b (if "Yes" to Intake Q10)
When were you vaccinated against flu this season (2011-2012)?

- XX/XX/XXXX
- I don't know/can't remember

Intake Q10c (if "Yes" to Intake Q10)
What were your reasons for getting a seasonal influenza vaccination this year? (Select all options that apply)

- I received a voucher at home
- I belong to a risk group, for which vaccination is recommended (pregnant women, people over 65, chronic disease condition, contact with children at risk and under 6 months, living in a long term care institution, obese, etc.)
- Vaccination decreases my risk of getting influenza
- Vaccination decreases the risk of spreading influenza to others
- My doctor recommended it
- It was recommended in my workplace/school
- The vaccine was readily available and vaccine administration was convenient
- The vaccine was free
- I don't want to miss work/school
- I always get the vaccine
- Other reason(s)

Intake Q10d (if "No" to Intake Q10, follow-up question)
What were your reasons for NOT getting a seasonal influenza vaccination this year? (Select all options that apply)

- I am planning to be vaccinated, but haven't been yet
- I haven't been offered the vaccine
- I don't belong to a risk group for which the vaccination is recommended (pregnant women, people over 65, chronic disease condition, contact with children at risk and under 6 months, living in a long term care institution, obese, etc.)
- It is better to build your own natural immunity against influenza
- I doubt that the influenza vaccine is effective
- Influenza is a minor illness
- I don't think that I am likely to get influenza
- I believe that influenza vaccine can cause influenza
- I am worried that the vaccine is not safe or will cause illness or other adverse events
- I don't like having vaccinations
- The vaccine is not readily available to me
- The vaccine is not free of charge
- No particular reason
- Although my doctor recommended a vaccine, I did not get one
- Other reason(s)

Intake Q11
Have you received a flu vaccine during the last autumn/winter season (i.e. 2010-2011)?*

- Yes
- No
- I don't know/can't remember

Intake Q12
Do you take regular medication for any of the following medical conditions?* (Select all options that apply)

- No, I do not have any of these diseases
- Asthma
- Diabetes
- Lung disorder (COPD, emphysema, …)

- Heart disorder
- Kidney disorder
- An immunocompromising condition (e.g. splenectomy, organ transplant, acquired immune deficiency, cancer treatment)

Intake Q13 (only asked of women between ages 15 and 50)
Are you currently pregnant?

- Yes [go to question Q13b]
- No [go to question Q14]
- Don't know/would rather not answer

Intake Q13b: (if "Yes" to Intake Q12)
Which trimester of the pregnancy are you in?

- First trimester (week 1-12)
- Second trimester (week 13-28)
- Third trimester (week 29-delivery)
- Don't know/would rather not answer

Intake Q14
Do you smoke tobacco?*

- No, I never smoked
- No, I stopped smoking more than one year ago
- No, I stopped smoking less than one year ago
- Yes, occasionally
- Yes, daily, fewer than 10 times a day
- Yes, daily, 10 or more times a day
- Don't know/would rather not answer

Intake Q15
Do you have one of the following allergies that can cause respiratory symptoms?* (Select all options that apply)

- Hay fever
- Allergy against house dust mite
- Allergy against domestic animals or pets
- Other allergies that cause respiratory symptoms (e.g. sneezing, runny eyes)
- I do not have an allergy that causes respiratory symptoms

Intake Q16
Do you follow a special diet? (Select all options that apply)

- No special diet
- Vegetarian
- Veganism
- Low-calorie
- Other

Intake Q17
Do you have pets at home? (Select all options that apply)

- No
- Yes, one or more dogs
- Yes, one or more cats
- Yes, one or more birds
- Yes, one or more other animals

Intake Q18
Where did you hear about the GrippeNet.fr project for the first time?

- On radio or television

- In the newspaper or in a magazine
- Via an Internet site (search engine or link)
- By the poster of the project
- Via family or friends
- Via school or work
- Via an healthcare professional
- A member of my family/friends work on this project

2. GrippeNet.fr intake survey (original French version).

Les questions obligatoires sont suivies d'un astérisque (*). Quand une seule réponse doit être donnée, les propositions sont précédées d'un point (•), quand plusieurs réponses peuvent être données, les propositions sont précédées d'un cercle (o).

Contexte Q0
Pour qui remplissez-vous ce questionnaire ? *
• Pour moi-même
• Pour un membre de mon foyer
• Pour quelqu'un d'autre
Si une réponse autre que « Pour moi-même » est cochée, la question Q0 bis est posée.

Contexte Q0b
Je certifie avoir expliqué à cette personne le contenu et les implications de cette étude, et avoir obtenu son libre consentement. Cette personne m'a donné pouvoir pour remplir les questionnaires à sa place. *
• Oui.
• Non
Si la case « non » est cochée, il n'est pas possible de poursuivre le remplissage du questionnaire. Il n'est également pas possible de remplir les autres questionnaires. Si la case oui est cochée, le texte suivant apparaît : « Répondez s-il-vous-plaît à toutes les questions comme si vous étiez la personne pour laquelle vous remplissez ce questionnaire.».

Contexte Q1
Quel est votre sexe ? *
• Homme
• Femme

Contexte Q2
Quelle est votre date de naissance (mois et année)? *
Si la date de naissance correspond à un individu âgé de moins de 18 ans, il n'est pas possible de poursuivre le remplissage du questionnaire, ni de remplir d'autres questionnaires.

Contexte Q3
Quel est le code postal de votre domicile ? *
Information non enregistrée dans la base de données, permettant uniquement de faciliter la réponse à la question suivante.

Contexte Q3b
Quelle est la commune de votre domicile ? *
(liste déroulante avec les communes correspondant au code postal donné en Q3)

Contexte Q4
Quelle est votre activité principale ? *

• Salarié, à temps plein
• Salarié, à temps partiel
• Travailleur indépendant (entrepreneur, commerçant, agriculteur, profession libérale…)
• Ecolier ou étudiant
• Homme ou femme au foyer
• Demandeur d'emploi
• En congé maladie longue durée ou en congé parental
• Retraité(e)
• Aucune de ces réponses

Contexte Q4b ***(si « emploi rémunéré », « travailleur indépendant » ou « écolier / étudiant » à Q4)***
Quel est le code postal de votre école / université / lieu de travail (là où vous passez la plus grande partie de votre temps à travailler/étudier)?
• XXXX
• Je ne sais pas / ne me souviens pas
• Non applicable (par exemple, je n'ai pas de lieu de travail fixe)
Information non enregistrée dans la base de données, permettant uniquement de faciliter la réponse à la question suivante.

Contexte Q4c ***(si code postal donné à Q4b)***
Quelle est la commune correspondante ?
(liste déroulante avec les communes correspondant au code postal donné en Q4b)

Contexte Q4d ***(si « emploi rémunéré » à Q4)***
Laquelle de ces propositions correspond le plus à votre activité principale :
• Cadre ou profession intermédiaire ne travaillant pas dans un bureau (médecin, professeur, infirmier…)
• Cadre ou profession intermédiaire travaillant dans un bureau (administration, comptabilité…)
• Profession dans le domaine du commerce, de la restauration, de l'hôtellerie, du loisir (vendeur, serveur, professeur de sport…)
• Ouvrier, artisan (mécanicien, électricien, technicien…)
• Autre métier manuel (agent d'entretien, agent de sécurité, chauffeur…)
• Autre

Contexte Q4e ***(question posée uniquement aux personnes âgées de 16 ans et plus)***
Quel est votre plus haut niveau de qualification ? Si vous êtes toujours scolarisé ou étudiant, cochez s'il-vous-plaît la case correspondant au diplôme le plus élevé que vous avez obtenu jusqu'à maintenant.
o Je n'ai aucun diplôme
o Diplôme national du brevet, CAP, BEP, CFPA…
o Baccalauréat, brevet professionnel, brevet de technicien…
o Licence, DUT, BTS…
o Master, diplôme d'ingénieur ou diplôme de troisième cycle (thèse d'université ou d'exercice…)
o Je suis toujours scolarisé ou étudiant

Contexte Q5
Avez-vous des contacts avec les personnes suivantes au cours d'une journée typique ? *
(Sélectionnez toutes les options qui s'appliquent, s'il y a lieu)
o Plus de 10 enfants ou adolescents dans une même journée
o Plus de 10 personnes âgées de plus de 65 ans dans une même journée
o Des personnes malades

o Des groupes de personnes (partage d’un même lieu avec plus de 10 individus en même temps, ex : dans un bureau)
o Aucune de ces réponses

Contexte Q6
Y compris vous, combien de personnes de chacune des classes d'âge suivantes vivent dans votre foyer ?
Je vis seul
0 - 4 ans
5 - 18 ans
19 - 44 ans
45 - 64 ans
65 ans et +

Contexte Q6b *(uniquement posée s’il y a au moins un enfant de 0-4 ans dans le foyer, y compris des participants)*
Combien d'enfants de 0 - 4 ans de votre foyer sont gardés en dehors du domicile (école, crèche, garderie, assistante maternelle…)?

Contexte Q6c (uniquement posée si réponse à la Q6b)
Dans quelle(s) structure(s) ce ou ces enfant(s) sont-ils gardés ?
o Ecole
o Assistante maternelle
o Garde partagée
o Crèche (collective, parentale, micro-crèche)
o Jardin d’enfant
o Crèche familiale
o Halte-garderie

Contexte Q7
Quel est votre principal moyen de déplacement ? *
• Marche
• Vélo
• Moto / scooter
• Voiture
• Transports en commun (bus, train, métro, etc.)
• Autre

Contexte Q7b
Lors d’une journée ordinaire, combien de temps passez-vous au total dans les transports en commun (bus, train, métro, etc.)? *
• Pas de temps du tout
• 0 - 30 minutes
• 30 minutes - 1 heure et demie
• 1 heure et demie - 4 heures
• Plus de 4 heures

Contexte Q8
A quelle fréquence avez-vous des rhumes ou la grippe ?
• Jamais
• Une ou deux fois par an
• Entre 3 et 5 fois par an
• Entre 6 et 10 fois par an

• Plus de 10 fois par an
• Je ne sais pas

Contexte Q9a
Avez-vous reçu un bon de vaccination contre la grippe cette année ? *
•Oui
•Non
•Je ne sais pas / ne me souviens pas

Contexte Q9b
Quelle est votre taille (en centimètres)?

Contexte Q9c
Quelle est votre poids (en kilogramme) ?

Contexte Q10
Avez-vous été vacciné contre la grippe cette année (depuis le mois de septembre dernier) ? * (en 2011-2012)
• Oui *[renvoi à Q10b et Q10c]*
• Non *[renvoi à Q10d]*
• Je ne sais pas / ne me souviens pas

Contexte Q10b:
Quand avez-vous été vacciné contre la grippe cette année (en 2011-2012)?
• XX / XX / XXXX
• Je ne sais pas / ne me souviens pas

Contexte Q10c:
Quelles étaient vos raisons pour vous faire vacciner contre la grippe cette année ? (Sélectionnez toutes les options qui s'appliquent)
o J'ai reçu un bon de vaccination à mon domicile
o Je fais partie d'un groupe à risque pour lequel la vaccination est recommandée (femmes enceintes, plus de 65 ans, problèmes de santé sous-jacents, contacts avec des enfants de moins de 6 mois à risque, résidents dans un établissement de soins de longue durée ou médico-social, obèses, etc.)
o La vaccination diminue mes risques de contracter la grippe
o La vaccination diminue le risque de transmettre la grippe à d'autres personnes
o Mon médecin me l'a recommandé
o Cela m'a été recommandé dans mon lieu de travail / école
o Le vaccin était aisément disponible et son administration était pratique
o Le vaccin était gratuit
o Je ne veux pas manquer le travail / l'école
o Je me fais toujours vacciner
o Autre(s) raison(s)

Contexte Q10d
Pourquoi n'avez-vous pas été vacciné contre la grippe saisonnière cette année ? (Sélectionnez toutes les options qui s'appliquent)
o Je prévois de me faire vacciner, mais je ne l'ai pas encore fait
o La vaccination ne m'a pas été proposée
o Je n'appartiens pas à un groupe à risque pour lequel la vaccination est recommandée (femmes enceintes, plus de 65 ans, problèmes de santé sous-jacents, contacts avec des enfants de moins de 6 mois à risque, résidents dans un établissement de soins de longue durée ou médico-social, obèses, etc.)

o Il est préférable de se construire sa propre immunité naturelle contre la grippe
o Je doute que le vaccin contre la grippe soit efficace
o La grippe est une maladie bénigne
o Je ne pense pas que je sois susceptible de contracter la grippe
o Je pense que le vaccin contre la grippe peut causer la grippe
o Je crains que le vaccin ne soit pas sûr ou cause une maladie ou d'autres événements indésirables
o Je n'aime pas recevoir de vaccins
o Le vaccin n'est pas facilement disponible pour moi
o Le vaccin n'est pas gratuit
o Je ne sais pas
o La vaccination m'a été recommandée par mon médecin, mais je ne me suis pas fait vacciner
o Autre(s) raison(s)

Contexte Q11
L'an dernier, avez-vous été vacciné contre la grippe ? * (en 2010-2011)
• Oui
• Non
• Je ne sais pas / ne me souviens pas

Contexte Q12
Prenez-vous régulièrement des médicaments pour l'un des problèmes médicaux suivants ? *
(Sélectionnez toutes les options qui s'appliquent)
o Non, je n'ai aucune des maladies qui figurent ci-dessous
o Asthme
o Diabète
o Troubles pulmonaires (BPCO, emphysème...)
o Troubles cardiaques
o Troubles rénaux
o Immunodépression (splénectomie, greffe d'organe, immunodéficience acquise, traitement de cancer en cours, prise de corticoïde ou d'un traitement immunosuppresseur…)

Contexte Q13 ***(posée uniquement aux femmes entre 15 et 50 ans)***
Êtes-vous enceinte ?
• Oui *[renvoi à Q12b]*
• Non *[renvoi à Q13]*
• Je ne sais pas / préfère ne pas répondre *[renvoi à Q13]*

Contexte Q13b:
A quelle trimestre de la grossesse êtes-vous ?
• Premier trimestre (semaines 1-12)
• Deuxième trimestre (semaines 13 - 28)
• Troisième trimestre (à partir de 29 semaines)
• Je ne sais pas / préfère ne pas répondre

Contexte Q14
Fumez-vous ? *
• Non, je n'ai jamais fumé
• Non, j'ai arrêté de fumer depuis plus d'un an
• Non, j'ai arrêté de fumer depuis moins d'un an
• Oui, occasionnellement
• Oui, tous les jours, moins de 10 cigarettes par jour
• Oui, tous les jours, 10 cigarettes ou plus par jour

• Je ne sais pas / préfère ne pas répondre

Contexte Q15
Avez-vous l'une des allergies respiratoires suivantes ? * (Sélectionnez toutes les options qui s'appliquent)
o Rhume des foins
o Allergie aux acariens
o Allergie aux animaux domestiques
o Autres allergies provoquant des symptômes respiratoires (par exemple des éternuements, des yeux larmoyants)
o Je n’ai pas d’allergie causant des symptômes respiratoires

Contexte Q16
Suivez-vous un régime alimentaire particulier ? (Sélectionnez toutes les options qui s'appliquent)
o Pas de régime alimentaire particulier
o Végétarien
o Végétalien
o Faible en calories
o Autre

Contexte Q17
Avez-vous des animaux à la maison ? (Sélectionnez toutes les options qui s'appliquent)
o Non
o Oui, un ou plusieurs chiens
o Oui, un ou plusieurs chats
o Oui, un ou plusieurs oiseaux
o Oui, un ou plusieurs autres animaux

Contexte Q18
Comment avez-vous eu connaissance de l’étude GrippeNet.fr ?
o Par la télévision ou la radio
o Par un journal ou magazine
o Par un site internet (moteur de recherche ou lien)
o Par le poster du projet
o Par un membre de ma famille ou un ami
o A l’école ou au travail
o Par un professionnel de la santé
o Par un de mes proches qui travaille sur le projet

3. Indicators non-comparable with national data

Table S1: Additional indicators collected in the Intake survey that cannot be compared to national statistics because of absence of data.

Indicators	Definition	Mean value (SD) or % in GrippeNet.fr population
Socio-demographic indicators		
Main occupation type (for paid employed or self employed persons) ★	Office work	57.1%
	Professional	26.3%
Daily contacts with specific groups of persons	With ≥10 children	12.5%
	With ≥10 elderly (65+)	10.4%
	With ≥10 persons	44.5%
Children in the household going to day-care?	Number of children aged [0-4] going to day-care	1.1 (0.7)
Main mean of transportation	Walking	14.0%
	Bike	4.3%
	Motorbike/scooter	1.6%
	Other	0.5%
Daily time in public transports	No time at all	69.9%
	[0-30’]	12.9%
	[30’-1h30’]	12.9%
	[1h30’-4h]	4.0%
	>4h	0.3%
How did you know about the study? ★	Via an internet site (search engine or link)	61.7%
	On radio or television	14.6%
Health-related indicators		
Frequency of flu/cold	Never	12.4%
	1-2 times/y	64.5%
	3-5 times/y	17.5%
	6-10 times/y	2.4%
	>10 times/y	0.9%
	Don’t know	2.2%
Date of vaccination	Time since the start date of the vaccination campaign in the fall 2011 (30/09/11), expressed in weeks	5.8 (3.8)
Reasons for vaccination during current season★	Vaccination decreases my risk of getting influenza	64.1%
	I always get the vaccine	52.1%
Reasons for non-vaccination during current season★	I don't belong to a risk group	52.4%
	I haven't been offered the vaccine	29.5%
Taking regular medications for risk conditions	Lung disorder	1.1%
	Heart disorder	8.0%

	Kidney disorder	0.2%
	Immunocompromising condition	1.6%
Pregnancy status	For women aged <50y	2.9%
Which trimester in pregnancy?	First trimester	35.9%
	Second trimester	20.6%
	Third trimester	43.5%
Respiratory allergies		34.9%
Special diet[1]		11.9%

★multiple answers to the question were possible; we only indicate the two reasons with largest responses
[1] include vegetarian - vegan, low-calorie, other

4. GrippeNet.fr participation

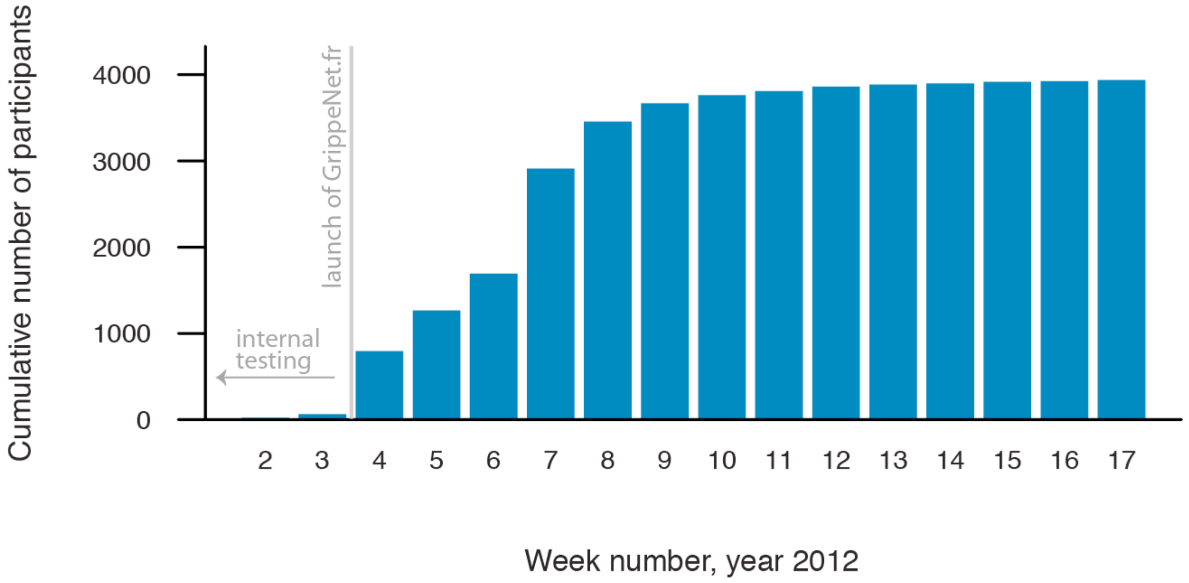

Figure S1: Behavior in time of the number of Grippenet.fr participants (individuals who filled ≥ 1 intake survey) during the first season.

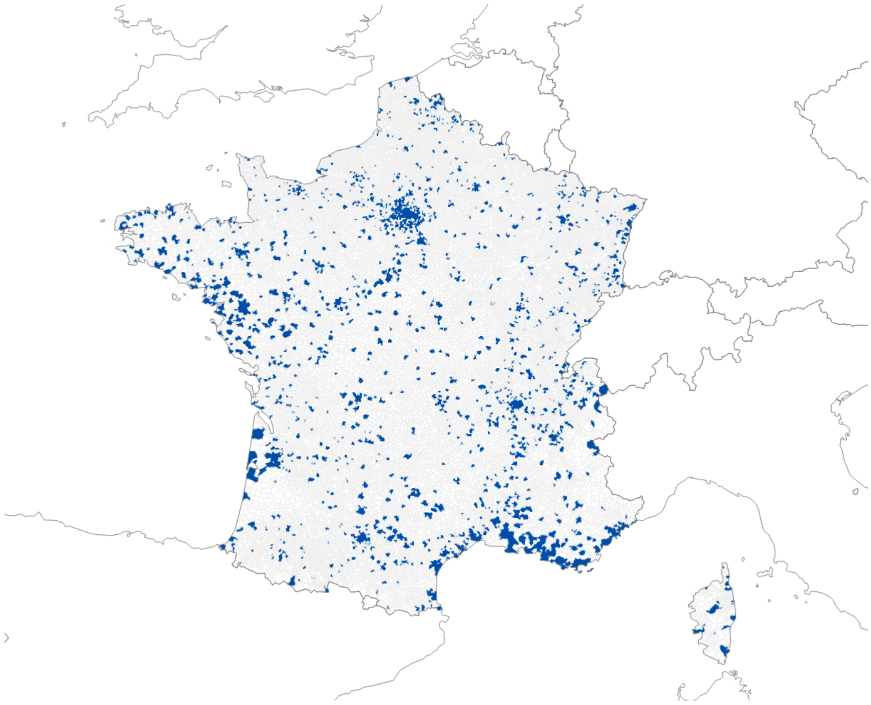

Figure S2. Municipalities with at least one person registered on GrippeNet.fr at the end of the season.

5. Commuting networks

5.1 Adjustment of census commuting datum

To account for possible discrepancies in the geographic distributions of GrippeNet.fr population with respect to the national data, the weight obtained from the national commuting source was corrected for bias in the population distribution by applying the rescaling with the ratio $\frac{N_O^{GN}}{N_O^{Fr}}$ between the GrippeNet.fr and the census population of the region of origin (respectively N_O^{GN} and N_O^{Fr}). This allowed a direct comparison of the weighted census data with the corresponding links obtained in the GrippeNet.fr sample.

5.2 Extraction of the Backbone of the commuting network

In order to extract the most relevant backbone of the census commuting network to be compared with the GrippeNet.fr network, we applied the disparity filter algorithm (1) that selects the links displaying statistically significant deviations with respect to a null model for the local assignment of weights to links. The null model used to define anomalous fluctuations is based on the null hypothesis that normalized weights are produced by a random assignment from a uniform distribution. Thus the disparity filter method selects the links, which deviate from the null hypothesis with a certain level of statistical significance α. The choice of the level of significance varies according to the purpose of the study and the properties of the network (1). In or case values of α in the range [0.005, 0.05] were all equivalent in selecting a minimal network (i.e. 10-15% of the links) including a large

proportion of the total number of commuters (60-75%). These α values also yielded equivalent results in terms of overlap between the filtered network and the GrippeNet.fr one. In Figure 4 we show the case with $\alpha = 0.01$.

5.3 Probability of observing a link in the GrippeNet.fr commuting network

The probability of a link in the census commuting network to be represented in the GrippeNet.fr network is computed as follow. Given the number of commuters w_{OD} along a given OD direction in the census data, the probability that an individual living in O would commute to D is given by $p_{OD} = w_{OD} / N_O^{Fr}$. If we take into account the sample N_O^{GN}, i.e. the number of individuals living in region O in the GrippeNet.fr population, and we assume the events to be independent, the probability that a participant of GrippeNet.fr living in O would commute to region D is given by

$$P_{OD} = 1 - (1 - p_{OD})^{N_O^{GN}} \quad \text{(S1)}$$

In Figure 3, the number of links in the census network associated to each probability interval is plotted against the percentage of such links represented in the GrippeNet.fr network.

5.4 Probability distribution of the commuter fluxes in the census data

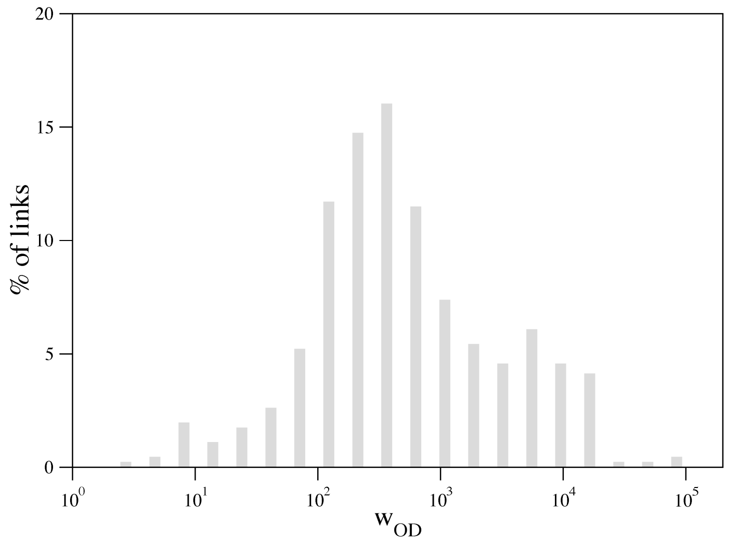

Figure S3. Probability distribution of the commuter fluxes, w_{OD}, in the census data.

5.5 Comparison of the incoming and outgoing fluxes

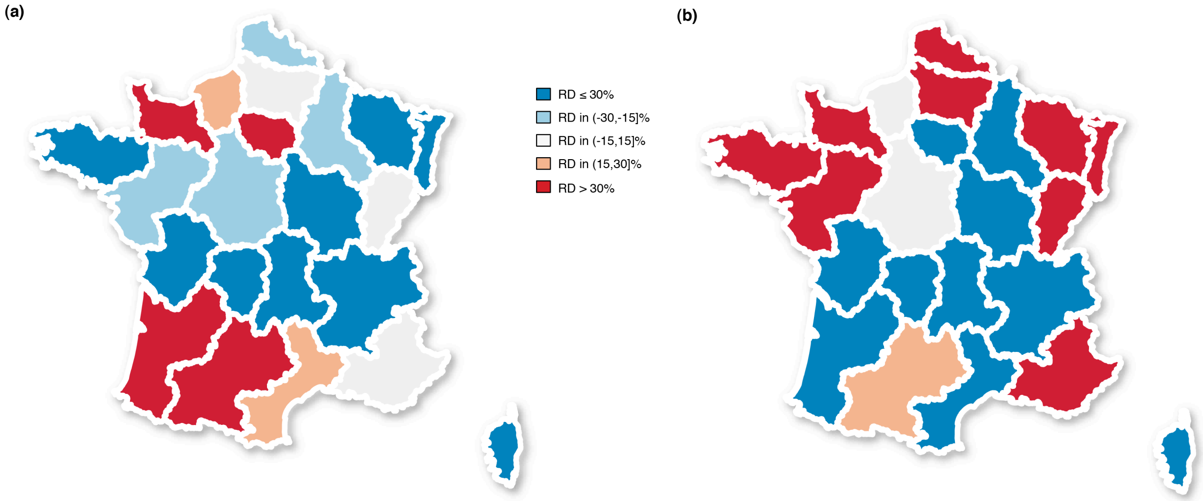

Figure S4. Comparison of the incoming and outgoing flows of commuters per region between GrippeNet.fr and the French population. a) Incoming flows per region. b) Outgoing flows per region. The color code indicates the relative difference (RD) between the regional distribution of the GrippeNet.fr flows of commuters and of the French census flows of commuters.

6. Influenzanet coverage

Table S2. Influenzanet activity and coverage per country in the 2011/2012 season (Influenzanet private communication). Countries are ranked by active participation rate.

country	registered users	active users	% of active users on the total	active participation rate (per 100,000)
NL	14479	12699	88%	76.2
BE	4362	3915	90%	35.5
SE	2657	1676	63%	17.8
PT	1410	1075	76%	10.2
FR	3936	3044	77%	6.2
UK	2547	1806	71%	2.9
IT	2283	1266	55%	2.1

References

1. Serrano MA, Boguna M, Vespignani A. Extracting the multiscale backbone of complex weighted networks. Proc Natl Acad Sci U S A. 2009 Apr 21;106(16):6483-8.
